# Comparison of the obturation quality of C-shaped canals via different filling techniques and ultrasonic vibration: Sealer-based obturation with ultrasonic vibration resulted in better filling quality in mandibular molars with C-shaped canals. Therefore, it can be an effective option for the treatment of C-shaped canals

**DOI:** 10.1186/s12903-025-07601-y

**Published:** 2026-01-20

**Authors:** Goun Bae, Young-Eun Jang, Yemi Kim

**Affiliations:** Department of Conservative Dentistry, College of Medicine, Ewha Womans University, 1071, Anyangcheon-ro, Yangcheon-gu, Seoul, 07985 Korea

**Keywords:** Obturation quality, C-shaped canal, Mandibular molar, Calcium silicate sealer, Ultrasonic, Filling techniques

## Abstract

**Introduction:**

The goal of this study was to compare the filling quality of C-shaped mandibular molars via microcomputed tomography (micro-CT) with that of sealer-based obturation technique (SBO), sealer -based obturation with ultrasonic vibration (SBU) and the continuous wave technique (CWT).

**Methods:**

Thirty mandibular molars with C-shaped canals were studied and assigned into three groups according to the filling method used. The CWT group was a continuous wave technique with a resin-based sealer; the SBO group was a sealer-based obturation technique with a calcium silicate sealer; and the SBU (sealer-based ultrasonic vibration) group was subjected to the same method as the SBO group with the addition of ultrasonic vibration. The teeth were scanned via micro-CT 2 times: after preparation and after root canal filling. The total void volume, open porosity and closed porosity were measured. The data were analyzed via ANOVA and Tukey’s test.

**Result:**

Compared with the other two groups, the SBU group presented a significantly smaller void volume. In the apical third, the SBU group presented a significantly lower mean percentage of total voids than did the CWT group. In the middle third, the SBU group presented a significantly smaller void volume than the other two groups did, and the percentage volume of voids in the CWT and SBO groups was similar. In the coronal third, no statistically significant differences were observed among the three obturation techniques. In all the obturation techniques, the open porosity was greater than the closed porosity, and the open porosity yielded results similar to those of the total voids.

**Conclusion:**

Sealer-based obturation with ultrasonic vibration resulted in a smaller void volume in mandibular molars with C-shaped canals. The results of this study suggest that sealer-based obturation with ultrasonic vibration can be an effective treatment option for C-shaped root canals.

## Introduction

The C-shaped root canal is an anatomical variation that involves connecting root canals with pins or web-like structures to form a C shape [1]. C-shaped root canals are common in mandibular second molars [1, 2], and their prevalence has been reported to range from 2.7% to 44.9% [3, 4], particularly high in Asian populations [1, 5, 6]. C-shaped canals are also found in maxillary molars and premolars [7]. In the C- shaped root canal, the canal configuration changes according to the root level [6] and there are long and narrow areas, such as the isthmus. Because of these irregularities and complexities, effective shaping, cleaning, and obturation are difficult, potentially leading to reinfection within the canal [8, 9].

The continuous wave technique (CWT) involves vertically pressing the gutta-percha at the apical third and then injecting heated gutta-percha and compressing it to fill the remaining part. This method is known to achieve good filling, especially in irregular or oval -shaped root canals [10–12]. However, there may be narrow areas in the C-shaped canal where it is difficult for the GP to enter [13, 14]. The sealer-based obturation technique (SBO) uses gutta-percha and calcium silicate sealers and fills unfilled areas with a sealer [15, 16]. Compared with CWT, SBO is considered less technique sensitive and has shown similar or superior filling quality [17, 18]. However, large amounts of sealer may increase porosity [19].

Ultrasonic vibration was suggested to improve the filing quality [20–22]. Ultrasonic vibration generates compressive forces and rearranges cement particles what leads to escape trapped air [23]. Some studies have reported that ultrasonic vibration may increase void formation due to excessive ultrasonic energy and affect the filling quality [24, 25]. Therefore, an indirect method allowing ultrasonic vibration to be transmitted to the sealer through the GP cone was proposed [23].

In the C-shaped root canal, the canal configuration changes according to the root level [6], and various anatomical complexities exist. There are difficulties in effective shaping, cleansing, and obturation. Previous studies examining root canal filling quality have mostly evaluated premolars [23, 26–29]. Therefore, the purpose of this study was to compare the filling quality of sealer-based obturation (SBO) and continuous wave technique (CWT) on real human teeth with C-shaped canals via micro-CT. In addition, we further investigated the effect of ultrasonic vibration on filling quality.

## Materials and methods

### Sample preparation

Among patients who visited the dental clinic of ewha womans university, teeth extracted for orthodontic or periodontal treatment were used in the experiment. A total of thirty human mandibular molars with C-shaped canals were included. The study was performed in accordance with the Declaration of Helsinki. Informed consent was obtained from all participants involved in the study. The protocol of this study was approved by the Institutional Review Board (IRB) of our institution (IRB number: EUMC 2023-03-009).

Teeth exhibiting decay, cracks, perforations, internal or external resorption, or those that had previously undergone root canal treatment were not included. The crown portion of each tooth was sectioned at the cemento-enamel junction.

Once the canal orifices were founded, a #10 K-file (Dentsply Maillefer, Ballaigues, Switzerland) was inserted into each canal until it was visible at the apical foramen, ensuring patency. After measuring the length, the working length was determined by subtracting 0.5 mm. After filling the canal with a 2.5% sodium hypochlorite (NaOCl), shaping was performed using ProTaper Next Ni-Ti rotary system (Dentsply Sirona Endodontics, Ballaigues, Switzerland), and Ni-Ti was used up to F3. After that, the canals were irrigated with 17% ethylenediaminetetraacetic acid (EDTA) for 1 min, washed with distilled water, irrigated with 5.25% NaOCl for 5 min, and washed again with distilled water. The canals were then dried with paper points.

### Root canal obturation

The total volume of the root canal was measured using micro CT. The teeth were categorized into three groups based on the total canal volume and then randomly obturated with different filling techniques. The filling method is as follows: the CWT group (continuous wave technique with AH Plus sealer), the SBO group (sealer-based obturation with EndoSeal MTA sealer), and the SBU group (SBO combined with ultrasonic vibration).

In the CWT group, AH plus sealer (Dentsply DeTrey, Konstanz, Germany), a resin-based sealer, was used. The gutta-percha (GP) cone coated with AH plus sealer was placed inside the canal, the GP cone was cut at a point 3 mm short of working length, and backfilling was performed using SuperEndo-Beta (B&L Biotech USA, Bala Cynwyd, PA).

In the SBO group, EndoSeal MTA sealer (Maruchi, Wonju, Korea), a calcium silicate-based sealer, was used. EndoSeal MTA sealer was injected into the root canal, and a GP cone was placed into the root canal and cut at the orifice level.

In the SBU group, EndoSeal MTA sealer was injected into the root canal, and a GP cone was placed into the root canal. After holding the GP cone with cotton plier, an ultrasonic tip (EMS, Nyon, Switzerland) was placed on the cotton plier, and 24 kHz ultrasonic vibration was applied for 1–2 s. The GP cone was cut at the orifice level.

To minimize potential air entrapment during GP cone insertion, the cones were inserted slowly and carefully without additional pumping or vertical manipulation, ensuring consistency across all experimental samples. During obturation procedures, care was taken to avoid sealer extrusion beyond the apical foramen, and no obvious apical extrusion was observed in any specimen.

The access cavities were sealed with temporary filling materials (caviton, GC Dental Industrial Corp., Tokyo, Japan) and the teeth were stored for seven days in a saline solution.

### Micro-CT evaluation

All the teeth in the three groups (*n* = 10) were scanned via a micro-CT system (SKYSCAN 1272, Bruker microCT, Kontich, Belgium) at 80 kV, 125 µA, a 1.0 mm aluminum filter, 180° rotation, a 0.6° rotation step and a frame average of 4, resulting in a pixel size of 12 μm. Images were reconstructed via Data Viewer 64 software (version 1.5.2.4, Bruker), and CT-An software (version 1.16.1.0, Bruker) was applied to analyze void volumes.

Voids were defined as empty spaces within the root canal that were not filled with gutta-percha or sealer. Among them, open porosity was classified as the empty space between the dentin wall and the gutta-percha or sealer, whereas closed porosity referred to the voids that did not contact the dentin wall and were located within the sealer itself or between the gutta-percha and sealer.

Cross sectional images perpendicular to the long axis of the root were acquired, and the images were converted to binary images via image thresholding. The areas of gaps and voids from the apex to the 9 mm level of each axial tooth slice were evaluated. To analyze the 3-dimensional volume of voids, binary images were used. A region of interest was set on each cut, and the percentage of voids within the canal was calculated. Three-dimensional analysis of the whole canal was performed, and calculations were performed at the apical (0–3 mm), middle (3–6 mm), and coronal (6–9 mm) levels.

Blinding was applied during outcome assessment. All micro-CT scans and void analyses were performed by a single examiner who was blinded to the experimental group assignments to minimize potential bias.

### Statistical analysis

The data were statistically analyzed via the Kolmogorov‒Smirnov test to determine a normal distribution, and then via one-way analysis of variance and Tukey tests to determine any significance (*P* < 0.05). All the statistical analyses were performed with SPSS software (version 25, SPSS Inc. Chicago, IL).

## Results

Table 1 shows the average percentage of the total void volume according the whole canal and according the different root thirds. In the whole canal, the mean percentage volume of total voids was 6.84% ± 1.35% (CWT), 7.00% ± 2.17% (SBO) and 4.43% ± 1.12% (SBU). Compared with the other two groups, the SBU group presented a lower void volume percentage (*P* < 0.05). In the apical third, the SBU group was associated with a significantly lower mean percentage of total voids (5.46% ± 1.33%) compared with the CWT group (9.54% ± 1.77%) (*P* < 0.05). In the middle third, the SBU group presented a significantly smaller void volume than the other two groups (*P* < 0.05). The percentage volume of voids associated with the CWT and SBO groups was similar. There were no significant differences among the three obturation techniques regarding the coronal third (*P* > 0.05).

**Table 1:** Percentage volume of total voids after obturation in the whole Canal

	Percentage volume of total voids (%)		
	CWT	SBO	SBU
Whole	6.84 ± 1.35^a^	7.00 ± 2.17^a^	4.43 ± 1.12^b^
Apical	9.54 ± 1.77^a^	7.59 ± 3.50^ab^	5.46 ± 1.33^b^
Middle	8.48 ± 2.64^a^	7.75 ± 2.40^a^	4.13 ± 1.23^b^
Coronal	5.97 ± 1.49^a^	6.35 ± 2.91^a^	4.25 ± 1.31^a^

Percentage volume of total voids (%) = volume of total voids / volume of entire root canal volume

Values are shown as the mean ± standard deviation. Different lowercase letters on the same row represent significant differences (*p* < 0.05)

*CWT* continuous wave technique, *SBO* sealer-based obturation technique, *SBU* sealer-based obturation technique with ultrasonic activation

Table 2 shows the percentage volumes of closed, open, and total porosities in the whole canal and each root third. In all the obturation techniques, the open porosity was greater than the closed porosity, and the open porosity yielded results similar to those of the total voids. In the apical third, the SBU group presented a significantly lower mean percentage of open porosities (4.75% ± 1.43%) than the CWT group (9.05% ± 1.70%) (*P* < 0.05).

**Table 2:** Percentage volumes of closed, open, and total porosities in the whole Canal

(a) Whole			
	Percentage volume of porosity (%)		
	CWT	SBO	SBU
Closed porosity (%)	0.51 ± 0.21^a^	0.58 ± 0.24^a^	0.57 ± 0.23^a^
Open porosity (%)	6.33 ± 1.31^a^	6.42 ± 2.11^a^	3.86 ± 0.99^b^
Total porosity (%)	6.84 ± 1.35^a^	7.00 ± 2.17^a^	4.43 ± 1.12^b^

**(b) Apical**			
	Percentage volume of porosity (%)		
	CWT	SBO	SBU
Closed porosity (%)	0.49 ± 0.26^a^	0.46 ± 0.20^a^	0.71 ± 0.44^a^
Open porosity (%)	9.05 ± 1.70^a^	7.13 ± 3.44^ab^	4.75 ± 1.43^b^
Total porosity (%)	9.54 ± 1.77^a^	7.59 ± 3.50^ab^	5.46 ± 1.33^b^

**(c) Middle**			
	Percentage volume of porosity (%)		
	CWT	SBO	SBU
Closed porosity (%)	0.59 ± 0.21^a^	0.63 ± 0.30^a^	0.64 ± 0.24^a^
Open porosity (%)	7.88 ± 2.51^a^	7.12 ± 2.33^a^	3.49 ± 1.27^b^
Total porosity (%)	8.48 ± 2.64^a^	7.75 ± 2.40^a^	4.13 ± 1.23^b^

(d) Coronal			
	Percentage volume of porosity (%)		
	CWT	SBO	SBU
Closed porosity (%)	0.48 ± 0.28^a^	0.59 ± 0.26^a^	0.52 ± 0.29^a^
Open porosity (%)	5.49 ± 1.53^a^	5.76 ± 2.93^a^	3.73 ± 1.22^a^
Total porosity (%)	5.97 ± 1.49^a^	6.35 ± 2.91^a^	4.25 ± 1.31^a^

Values are shown as the mean ± standard deviation. Different lowercase letters on the same row represent significant differences in porosity volume depending on different filling technique (*p* < 0.05)

**Fig. 1:**
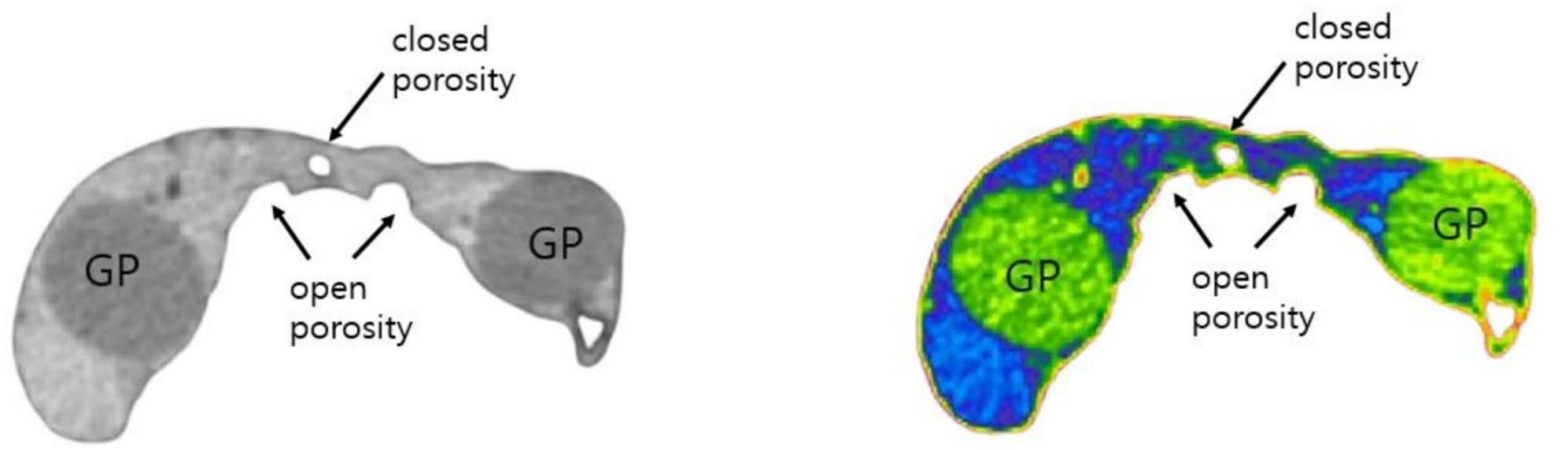
Representative sections from micro-CT imaging illustrating the cross section of a root canal filling

In the middle third, the SBU group presented less voids than the other groups (*P* < 0.05) In the cervical third, there were no statistically significant differences among the three filling techniques (*P* > 0.05). Closed porosity did not differ among the three obturation techniques (*P* > 0.05). Figure 1 shows micro-CT images illustrating the cross sectional images of a root canal filling.

## Discussion

Previous studies examining root canal filling quality have mostly evaluated premolars [23, 30–33]. To the best of our knowledge, no studies have evaluated filling quality via sealer-based obturation and ultrasonic vibration in mandibular molars with C-shaped root canals. A previous study [14] used a C-shaped canal resin model created via 3D printing; however, it only targeted the C1 configuration and hardly represented the entire C-shaped root canal. In this study, we evaluated obturation quality according to filling techniques for real human teeth with C-shaped canals. In addition, we aimed to improve the validity of the study by measuring the entire root canal volume and assigning groups based on similarity to eliminate important anatomic bias that could affect the results.

A previous study [34] investigated four epoxy resin-based sealers and revealed no differences in the percentage of voids when activated by ultrasonication. Ultrasonic vibration effectively increased the bond strength of the calcium silicate-based sealers, but the bond strength of the epoxy resin-based sealers did not differ before and after ultrasonic vibration. According to the results of a previous study, the AH plus used in CWT appears to be less affected by ultrasonic vibration. Therefore, additional ultrasonic vibration was performed only in SBO using EndoSeal MTA, a calcium silicate-based sealer.

The increase in porosity at the interface between the sealer and the dentinal wall can promote the growth and movement of bacteria to the periapical region, causing periapical periodontitis [35]. Therefore, it has more clinical impact than closed porosity. In this study, open porosity and closed porosity were investigated separately. The results revealed that the percentage of open porosity exceeded that of closed porosity, which was consistent with the findings of a previous study [36].

The goal of endodontic treatment is to remove bacteria and infected pulp via mechanical and chemical methods and then seal the root canal to prevent it from becoming infected again [37, 38]. To achieve this goal, various root canal obturation techniques and materials have been developed [39]. SBO has become popular because of its simplicity, low technique sensitivity, and filling quality, which is comparable to that of CWT. Previous studies comparing void volume between the continuous wave technique and sealer-based obturation technique reported that there was no difference between groups, which is consistent with the results of this study [17, 19, 29, 40].

In sealer-based obturation, the sealer fills the empty space between the GP cone and dentin. Ultrasonic vibration was proposed as a method to improve the filling quality of sealers. However, there are also reports that excessive ultrasonic vibration has a negative effect on void formation [22]. A previous study applied an indirect method that allows ultrasonic vibrations to pass through three media: an ultrasonic tip, a cotton plier, and a GP cone [23]. In this study, ultrasonic vibration was applied in the same way, and the results revealed that the percentage of voids was low in the SBU group.

The results of this study revealed better filling quality when ultrasonic vibration was applied. In previous studies comparing the filling quality among groups generated via the continuous wave technique, sealer-based obturation, and sealer-based obturation with ultrasonic vibration, no differences were detected among the 3 groups via micro-CT analysis [23]. These studies were conducted on maxillary premolars, which generally have relatively wide canals. In contrast, C-shaped canals feature narrow and complex canal anatomy, where ultrasonic activation is likely to have a more pronounced impact on sealer distribution and canal filling quality. Another study [19] reported better filling quality when ultrasound vibration was performed via the single cone technique, which is consistent with the results of this study. Ultrasonic vibration generates rapid compressive impulses, which reduce surface friction between cement particles and minimize voids.

One limitation of this study is the characteristics of the C-shaped root canals. Even in the same C-shaped root canal, the difficulty of root canal obturation depends on the anatomical complexity of the root canal. For example, the C1 and C2 configurations, with long and narrow isthmuses, complicate the shaping and acquisition of canals. To overcome this limitation, teeth were assigned to each group on the basis of premeasured canal volumes to compensate for the anatomical variation. However, further research is needed to standardize the C-shaped canal configurations.

## Conclusion

Sealer-based obturation with ultrasonic vibration resulted in a smaller void volume in mandibular molars with C-shaped canals. It can be an effective treatment option for C-shaped root canals on the basis of its better filling quality and lower technique sensitivity.

## Data Availability

The datasets used and/or analysed during the current study are available from the corresponding author on reasonable request.

## References

[1] Zheng Q, Zhang L, Zhou X, Wang Q, Wang Y, Tang L, et al. C-shaped root Canal system in mandibular second molars in a Chinese population evaluated by cone-beam computed tomography. Int Endod J. 2011;44(9):857–62. 21599707 10.1111/j.1365-2591.2011.01896.x

[2] Sidow SJ, West LA, Liewehr FR, Loushine RJ. Root Canal morphology of human maxillary and mandibular third molars. J Endod. 2000;26(11):675–8. 11469300 10.1097/00004770-200011000-00011

[3] Jin GC, Lee SJ, Roh BD. Anatomical study of C-shaped canals in mandibular second molars by analysis of computed tomography. J Endod. 2006;32(1):10–3. 16410060 10.1016/j.joen.2005.10.007

[4] Weine FS, Pasiewicz RA, Rice RT. Canal configuration of the mandibular second molar using a clinically oriented in vitro method. J Endod. 1988;14(5):207–13. 3251974 10.1016/S0099-2399(88)80171-7

[5] Yang ZP, Yang SF, Lin YC, Shay JC, Chi CY. C-shaped root canals in mandibular second molars in a Chinese population. Endod Dent Traumatol. 1988;4(4):160–3. 3267526 10.1111/j.1600-9657.1988.tb00315.x

[6] Kim SY, Kim BS, Kim Y. Mandibular second molar root Canal morphology and variants in a Korean subpopulation. Int Endod J. 2016;49(2):136–44. 25652228 10.1111/iej.12437

[7] De Moor RJ. C-shaped root Canal configuration in maxillary first molars. Int Endod J. 2002;35(2):200–8. 12019491 10.1046/j.1365-2591.2002.00461.x

[8] Melton DC, Krell KV, Fuller MW. Anatomical and histological features of C-shaped canals in mandibular second molars. J Endod. 1991;17(8):384–8. 1809802 10.1016/S0099-2399(06)81990-4

[9] Solomonov M, Paque F, Fan B, Eilat Y, Berman LH. The challenge of C-shaped Canal systems: a comparative study of the self-adjusting file and protaper. J Endod. 2012;38(2):209–14. 22244638 10.1016/j.joen.2011.10.022

[10] Weine FS. Endodontic therapy on the mandibular second molar: easiest to treat of the difficult, molar teeth. Compendium. 1994;15(9):1130. 3–6 passim; quiz 40. 7987904

[11] Simon JH. C-shaped canals: diagnosis and treatment. Gen Dent. 1993;41 Spec 482-5. 23087935

[12] Jerome CE. C-shaped root Canal systems: diagnosis, treatment, and restoration. Gen Dent. 1994;42(5):424–7. quiz 33 – 4. 7489874

[13] Matsune K. Molecular genetic study of the gutter shaped root (GSR) on mouse chromosome 17. J Oral Sci. 2000;42(1):21–6. 10808271 10.2334/josnusd.42.21

[14] Gok T, Capar ID, Akcay I, Keles A. Evaluation of different techniques for filling simulated C-shaped canals of 3-dimensional printed resin teeth. J Endod. 2017;43(9):1559–64. 28756962 10.1016/j.joen.2017.04.029

[15] Fan B, Cheung GS, Fan M, Gutmann JL, Bian Z. C-shaped Canal system in mandibular second molars: part I–Anatomical features. J Endod. 2004;30(12):899–903. 15564874 10.1097/01.don.0000136207.12204.e4

[16] Ordinola-Zapata R, Bramante CM, de Moraes IG, Bernardineli N, Garcia RB, Gutmann JL. Analysis of the gutta-percha filled area in C-shaped mandibular molars obturated with a modified microseal technique. Int Endod J. 2009;42(3):186–97. 19228207 10.1111/j.1365-2591.2008.01495.x

[17] Celikten B, Uzuntas CF, Orhan AI, Orhan K, Tufenkci P, Kursun S, Demiralp K. Evaluation of root Canal sealer filling quality using a single-cone technique in oval shaped Canals: an in vitro Micro-CT study. Scanning. 2016;38(2):133–40. 26228657 10.1002/sca.21249

[18] Ersahan S, Aydin C. Solubility and apical sealing characteristics of a new calcium silicate-based root Canal sealer in comparison to calcium hydroxide-, methacrylate resin- and epoxy resin-based sealers. Acta Odontol Scand. 2013;71(3–4):857–62. 23088627 10.3109/00016357.2012.734410

[19] Kim SY, Jang YE, Kim BS, Pang EK, Shim K, Jin HR et al. Effects of ultrasonic activation on root Canal filling quality of Single-Cone obturation with calcium Silicate-Based sealer. Mater (Basel). 2021;14(5):1292. 10.3390/ma14051292 PMC7962949 33800442

[20] Lawley GR, Schindler WG, Walker WA 3rd, Kolodrubetz D. Evaluation of ultrasonically placed MTA and fracture resistance with intracanal composite resin in a model of apexification. J Endod. 2004;30(3):167–72. 15055436 10.1097/00004770-200403000-00010

[21] Yeung P, Liewehr FR, Moon PC. A quantitative comparison of the fill density of MTA produced by two placement techniques. J Endod. 2006;32(5):456–9. 16631848 10.1016/j.joen.2005.08.008

[22] Parashos P, Phoon A, Sathorn C. Effect of ultrasonication on physical properties of mineral trioxide aggregate. Biomed Res Int. 2014;2014:191984. 24800211 10.1155/2014/191984 PMC3988719

[23] Kim JA, Hwang YC, Rosa V, Yu MK, Lee KW, Min KS. Root Canal filling quality of a premixed calcium silicate endodontic sealer applied using Gutta-percha Cone-mediated ultrasonic activation. J Endod. 2018;44(1):133–8. 29102078 10.1016/j.joen.2017.07.023

[24] Aminoshariae A, Hartwell GR, Moon PC. Placement of mineral trioxide aggregate using two different techniques. J Endod. 2003;29(10):679–82. 14606796 10.1097/00004770-200310000-00017

[25] El-Ma’aita AM, Qualtrough AJ, Watts DC. A micro-computed tomography evaluation of mineral trioxide aggregate root Canal fillings. J Endod. 2012;38(5):670–2. 22515899 10.1016/j.joen.2012.01.009

[26] Shemesh H, van den Bos M, Wu MK, Wesselink PR. Glucose penetration and fluid transport through coronal root structure and filled root canals. Int Endod J. 2007;40(11):866–72. 17877722 10.1111/j.1365-2591.2007.01302.x

[27] Del Fabbro M, Corbella S, Sequeira-Byron P, Tsesis I, Rosen E, Lolato A, Taschieri S. Endodontic procedures for retreatment of periapical lesions. Cochrane Database Syst Rev. 2016;10(10):Cd005511. 27759881 10.1002/14651858.CD005511.pub3 PMC6461161

[28] An HJ, Yoon H, Jung HI, Shin DH, Song M. Comparison of obturation quality after MTA orthograde filling with various obturation techniques. J Clin Med. 2021;10(8):1719. 10.3390/jcm10081719 PMC8074131 33923426

[29] Somma F, Cretella G, Carotenuto M, Pecci R, Bedini R, De Biasi M, Angerame D. Quality of thermoplasticized and single point root fillings assessed by micro-computed tomography. Int Endod J. 2011;44(4):362–9. 21255040 10.1111/j.1365-2591.2010.01840.x

[30] Ko SY, Choi HW, Jeong ED, Rosa V, Hwang YC, Yu MK, Min KS. Main and accessory Canal filling quality of a premixed calcium silicate endodontic sealer according to different obturation techniques. Mater (Basel). 2020;13:19. 10.3390/ma13194389 PMC7579472 33019753

[31] Penha da Silva PJ, Marceliano-Alves MF, Provenzano JC, Dellazari RLA, Gonçalves LS, Alves FRF. Quality of root Canal filling using a bioceramic sealer in oval canals: A Three-Dimensional analysis. Eur J Dent. 2021;15(3):475–80. 33535249 10.1055/s-0040-1722095 PMC8382469

[32] Tavares K, Pinto JC, Santos-Junior AO, Torres FFE, Guerreiro-Tanomaru JM, Tanomaru-Filho M. Micro-CT evaluation of filling of flattened root canals using a new premixed ready-to-use calcium silicate sealer by single-cone technique. Microsc Res Tech. 2021;84(5):976–81. 33278309 10.1002/jemt.23658

[33] Roizenblit RN, Soares FO, Lopes RT, Dos Santos BC, Gusman H. Root Canal filling quality of mandibular molars with endosequence BC and AH plus sealers: A micro-CT study. Aust Endod J. 2020;46(1):82–7. 31556201 10.1111/aej.12373

[34] Guimarães BM, Amoroso-Silva PA, Alcalde MP, Marciano MA, de Andrade FB, Duarte MA. Influence of ultrasonic activation of 4 root Canal sealers on the filling quality. J Endod. 2014;40(7):964–8. 24935544 10.1016/j.joen.2013.11.016

[35] Gillen BM, Looney SW, Gu LS, Loushine BA, Weller RN, Loushine RJ, et al. Impact of the quality of coronal restoration versus the quality of root Canal fillings on success of root Canal treatment: a systematic review and meta-analysis. J Endod. 2011;37(7):895–902. 21689541 10.1016/j.joen.2011.04.002 PMC3815527

[36] Milanovic I, Milovanovic P, Antonijevic D, Dzeletovic B, Djuric M, Miletic V. Immediate and Long-Term porosity of calcium Silicate-Based sealers. J Endod. 2020;46(4):515–23. 32094001 10.1016/j.joen.2020.01.007

[37] Li GH, Niu LN, Zhang W, Olsen M, De-Deus G, Eid AA, et al. Ability of new obturation materials to improve the seal of the root Canal system: a review. Acta Biomater. 2014;10(3):1050–63. 24321349 10.1016/j.actbio.2013.11.015 PMC3939610

[38] Keleş A, Keskin C. Presence of voids after warm vertical compaction and single-cone obturation in band-shaped isthmuses using micro-computed tomography: A Phantom study. Microsc Res Tech. 2020;83(4):370–4. 31829476 10.1002/jemt.23423

[39] De-Deus G, Souza EM, Silva E, Belladonna FG, Simões-Carvalho M, Cavalcante DM, Versiani MA. A critical analysis of research methods and experimental models to study root Canal fillings. Int Endod J. 2022;55(Suppl 2):384–445. 35226760 10.1111/iej.13713

[40] Schäfer E, Nelius B, Bürklein S. A comparative evaluation of gutta-percha filled areas in curved root canals obturated with different techniques. Clin Oral Investig. 2012;16(1):225–30. 21249509 10.1007/s00784-011-0509-z

